# Modulatory Mechanism of Polyphenols and Nrf2 Signaling Pathway in LPS Challenged Pregnancy Disorders

**DOI:** 10.1155/2017/8254289

**Published:** 2017-08-23

**Authors:** Tarique Hussain, Bie Tan, Gang Liu, Ghulam Murtaza, Najma Rahu, Muhammad Saleem, Yulong Yin

**Affiliations:** ^1^National Engineering Laboratory for Pollution Control and Waste Utilization in Livestock and Poultry Production, Key Laboratory of Agro-Ecological Processes in Subtropical Region, Institute of Subtropical Agriculture Chinese Academy of Sciences, Changsha, Hunan 410125, China; ^2^University of the Chinese Academy of Sciences, Beijing 10008, China; ^3^Hunan Collaborative Innovation Center for Utilization of Botanical Functional Ingredients and Hunan Collaborative Innovation Center of Animal Production Safety, Changsha, Hunan 410000, China; ^4^Shaheed Benazir Bhutto University of Veterinary & Animal Sciences, Sakrand, Sindh 67210, Pakistan; ^5^Department of Veterinary Microbiology, Faculty of Animal Husbandry and Veterinary Sciences, Sindh Agriculture University, Tandojam, Sindh 70050, Pakistan; ^6^Food Engineering and Bioprocess Technology, Asian Institute of Technology, Bangkok 12120, Thailand

## Abstract

Early embryonic loss and adverse birth outcomes are the major reproductive disorders that affect both human and animals. The LPS induces inflammation by interacting with robust cellular mechanism which was considered as a plethora of numerous reproductive disorders such as fetal resorption, preterm birth, teratogenicity, intrauterine growth restriction, abortion, neural tube defects, fetal demise, and skeletal development retardation. LPS-triggered overproduction of free radicals leads to oxidative stress which mediates inflammation via stimulation of NF-*κ*B and PPAR*γ* transcription factors. Flavonoids, which exist in copious amounts in nature, possess a wide array of functions; their supplementation during pregnancy activates Nrf2 signaling pathway which encounters pregnancy disorders. It was further presumed that the development of strong antioxidant uterine environment during gestation can alleviate diseases which appear at adult stages. The purpose of this review is to focus on modulatory properties of flavonoids on oxidative stress-mediated pregnancy insult and abnormal outcomes and role of Nrf2 activation in pregnancy disorders. These findings would be helpful for providing new insights in ameliorating oxidative stress-induced pregnancy disorders.

## 1. LPS Overview and Its Drawbacks

**Figure 1:**
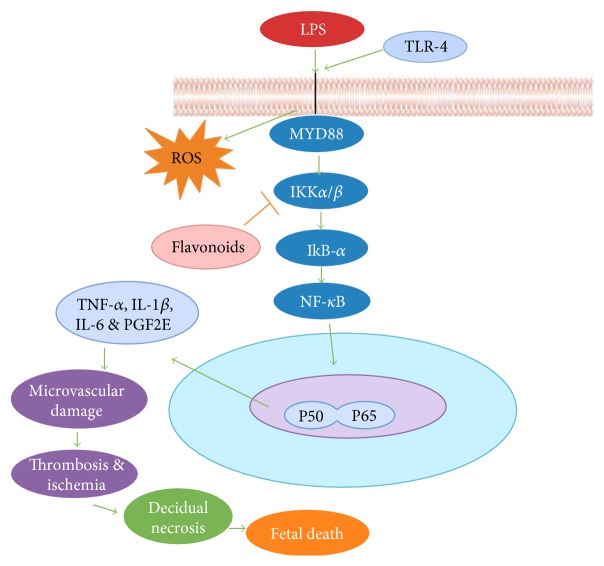
LPS-induced abortion by regulating strong cellular network. After induction of LPS, binding protein interacts with Toll-like receptor 4 (TLR4) and activates downstream adaptor proteins MYD-88, which subsequently stimulate IKK complex, resulting in ubiquitination and phosphorylation of IkB*α* proteins that translocate NF-*κ*B into the nucleus for production of several proinflammatory cytokines such as TNF-*α*, IL-*β*, IL-6, and PGF2E which causes microvascular damage leading to thrombosis, ischemia, necrosis of decidual cells, and finally abortion. On the other hand, flavonoids prevent abortion by inhibition of IKK complex proteins and bring NF-*κ*B into its inactivated form in cytoplasm. These beneficial effects of flavonoids are mediated by activation of PI3K/Akt pathway; hence, it prevents development of free radicals by supplementation of flavonoids during pregnancy.

Early pregnancy failure is a main obstacle that leads to significant effects on pregnancy outcomes in humans and animals [1]. Approximately 15–20% clinical pregnancies experience abortion [2], and about 30–50% conception resulted in early embryonic loss in mammals [3]. Moreover, assisted reproductive techniques enhance pregnancy rate in infertile women without avoiding early embryonic loss [4]. Humans get constant exposure of LPS at minimum levels in gastrointestinal inflammatory diseases [5]. Lipopolysaccharide (LPS) is derived from G-negative bacteria; maternal exposure to pregnant rodents causes placental inflammation contributing in embryonic resorption, fetal growth restriction (FGR), preeclampsia fetal brain injury, and miscarriages which develops by alternation in cytokine productions [6, 7]. These cytokines were released by trophoblastic, decidual, and chorioamnionitic cells and other cell types [8]. In humans, LPS infection provokes fetal loss and preterm labor [9] and is thought to be regulated by LPS-induced ROS-mediated teratogenesis [10]. In addition, basal amount of ROS is necessary in early embryonic growth and metabolism; excessive generation of uterine ROS is detrimental to oxidative DNA damage of the embryo [11, 12]. In pregnant mice, LPS exposure at late gestation leads to preterm delivery and fetal demise [13, 14], and in later gestational stages, maternal LPS infection causes intrauterine fetal growth restriction [15]. The signaling molecule, nitric oxide (NO), displays an essential role in implantation, decidualization, vasodilation, myometrial relaxation, and overactivation possibly induced by free radical-mediated pathology. Enhanced production of LPS-induced nitric oxide has been reported in embryonic resorption and abortion [16]. LPS-triggered abortion mechanism has been depicted in Figure 1(). Nrf2 proteins display a key role in the elimination of oxidative stress through Nrf2-ARE signaling pathway [17, 18] as reported in preeclampsia conditions [19]. Nrf2 is very sensitive to maternal immune status and is responsible for fetal growth and survival through maintaining fetus desirable placental environment; later, Nrf2 protein expression of the placenta was decreased following delivery [20] suggesting its important function in fetal survival. Thus, any inappropriate function could lead to inducing numerous pregnancy disorders. The flavonoids of the polyphenol group are well-recognized natural compounds, which elicit strong antioxidant and anti-inflammatory activities that would be helpful in the elimination of LPS-potentiated pregnancy disorders. The polyphenols such as curcumin possess strong anti-inflammatory activity through influencing diverse pathways to modulate cellular functions. It can also decrease inflammation by inhibiting NF-*κ*B pathway via inactivation of IKK complexes [21, 22]. A study reported that curcumin polyphenols suppress methylglyoxal-induced apoptosis in mouse ESC-B5 cells and blastocysts by inhibiting reactive oxygen species (ROS) [23]. The anti-inflammatory strategy would be helpful in alleviating pregnancy-related complications [24]. This review emphasizes LPS-mediated pregnancy disorders and adverse birth outcomes, modulatory activities of polyphenols, and the role of Nrf2 signaling pathway. We have given detailed description below on the previous reports of polyphenol supplementations such as epigallocatechin gallate, curcumin, baicalin, and tricin which attenuate LPS-induced reproductive disorders, while genistein and quercetin develop strong antioxidant pregnant uterine environment that encounters disease in extrauterine life. These findings would be helpful in improving animal productions.

### 1.1. Disruption Pregnant Uterine Environment by Inflammatory Cytokines

The LPS binds with Toll-like receptor 4 (TLR4) with the facilitation of cluster of differentiation 14 (CD14) on cell surface of macrophages and monocytes. Upon activation of TLR4, it disseminates LPS signals to myeloid differentiation factor (MYD88) adaptor molecules; thus, its stimulation is known to be regulated by several signaling molecules including NF-*κ*B proteins [25]. NF-*κ*B exerts an important role in the development of inflammation while its activation occurs by degradation and phosphorylation of I*κ*B kinases such as IKK*α* and IKK*β* which results in the translocation of NF-*κ*B into the nucleus where it induces the formation of inflammatory cytokines, tumor necrosis factor-*α* (TNF-*α*), interleukin-beta (IL-1*β*), interleukin-6 (IL-6), and interleukin-8 (IL-8), and inducible inflammatory enzymes, nitric oxide (NO) and reactive oxygen species (ROS) [26, 27]. As mentioned above, that LPS persuades inflammation which triggered various pregnancy disorders in mid and late gestation [28]. The inflammatory mediators, such as TNF-*α*, interrupt placental blood supply and its function [29] resulting in fetal injury [30]. Studies on mice report that inflammation mediated by TNF-*α* and interferon-gamma (IFN*γ*) in macrophages and uterine natural killer (uNK) cells causes vascular injury and placental ischemia in uterine endothelial cells [31]. It was further noted that LPS mediates IFN*γ* and TNF-*α* through activation of Toll-like receptors and is responsible for activation of cytokine-induced abortion [32] by possibly downregulating expression of cyclooxygenase-2 enzyme (COX-2) protein that encounters fibrinogen-like protein-2 (Fgl2) in the fetomaternal site [33]. The abortogenic effect varies according to the nature of LPS, source, time length, and dose regimen [32]. The interleukin-10 (IL-10) is an anti-inflammatory cytokine which minimizes pregnancy-related inflammation through regulation of TNF-*α* and other cytokines and chemokines [34]. The growing body of texture revealed that maternal LPS induced uterine inflammation by cytokines through transplacental transmission that enhances the risk of brain diseases at the adult stage of life [35].

The peroxisome proliferator-activated receptor (PPAR) is a nuclear protein stimulated by multiple ways such as activations of prostaglandins (PGs) and leukotrienes (LTs). After activation, it stimulates transcription factors and mediates various cellular functions including cell differentiation, apoptosis, lipid metabolism, peroxisome proliferation, and inflammation response. In pregnancy, PPAR signals regulate trophoblast invasion and differentiation [36], placentation [37], and maternal metabolism [38]. The improper regulation of PPAR receptors causes complications including preeclampsia (PE), intrauterine growth restriction (IUGR), and preterm birth [39]. *In vitro* studies on knockout mice propose that stimulation of PPAR suppresses proinflammatory cytokines and distinguishes immune cells from anti-inflammatory phenotypes [40]. Naturally occurring compounds polyphenols exert ability to stimulate PPAR nuclear receptors and exert fruitful impact on pregnancy. It has been noted that PPAR*γ* was considered as a potential target for therapeutic intervention against preeclampsia [41]. Limited research on PPAR signals in pregnancy disorders have been observed; therefore, more studies are needed to explore further insights.

## 2. Positive Effects of Cytokines in Pregnancy Development

Naturally, the immune system protects uterine environment from invading pathogens to full-term birth [42]. Excessive levels of endometrial cytokines, prostaglandins, and leukocytes are released during inflammatory condition [43]. The endometrial mediated cytokine and chemokine productions give directions to the blastocyst to connect with endometrial walls. When invasion and lysis of trophoblast exist, conversion from epithelial cells to stromal cells repairs endometrial tissue which replaces cells in the placenta. This structure is mediated by Th1 cellular responses where an ample amount of proinflammatory molecules such as IL-6, LIF, IL-8, and TNF-*α* was contributed [44] and these also recruit immune cells towards the decidua. In human and mouse, a huge population of decidual leukocytes has been witnessed at the site of implantation. Of note, these cells are comprised of 65–70% uterine natural killer (uNK) cells and 10–20%, based on macrophages and dendritic cells (DCs) [45]. The macrophages and DCs localize in the decidua during the entire pregnancy and exhibit a key role at maternal-fetal interface [46]. The macrophages and DCs have the capability to secrete a variety of anti-inflammatory molecules (IL-4, IL-10, and IL-13) and enzymes, which are mainly involved in structural modification and angiogenesis [47]. Moreover, it was documented that macrophages mediate trophoblast invasion and might exert main function in eliminating debris which comes from trophoblastic apoptosis at different phases of gestation [44]. The presence of DCs in maternal tissue during implantation has been observed [48], and it was further illustrated that DCs have the ability to alter Th1 proinflammatory cytokines to Th2 anti-inflammatory cytokines at latter stages of gestation [49]. Near to parturition, anti-inflammatory response converted into pro-inflammatory response in order to induce uterine contractions initiates to parturition [50]. Overall, observation indicates the key functions of anti- to pro-inflammatory cytokine response during the entire pregnancy. Of note, limited evidences of inflammatory response have been documented before implantation of the embryo.

## 3. Interruption in Redox Balance

In normal homeostasis, ROS are neutralized by antioxidant defense *in vivo*. This balance is encountered by overpowering of ROS production and incompetency of antioxidant system to eliminate them. Growing evidences show that early exposure of oxidative stress in pregnancy might have long-term complications [51]. The antioxidant defense against locally produced NO by inducible nitric oxide synthase enzyme downregulates NO signals in the placenta which are crucially important for normal vascular development. In the first trimester of pregnancy, fetal growth was subjected to hypoxia [52], while in the prenatal period, it was documented that the fetus is highly vulnerable to oxidative damage whereas antioxidant supplementation during pregnancy ameliorates reproductive disorders such as implantation failure and fetal anomalies [53]. It has been reported that enhanced sodium dismutase-1 (SOD1) in mice suppresses fetal anomalies and protects against diabetes-related embryopathy [54]. In pregnancy, having intrauterine growth restriction (IUGR), preeclampsia (PE), and gestational diabetes mellitus (GDM) has been recorded to have higher chances of fetal hypoxia (markers of oxidative stress). Moreover, a deficient supply of oxygen has been observed in pathogenesis of IUGR and PE conditions [55]; on the other hand, preterm delivery arises from ischemia-reperfusion injury which decreased body weight.

## 4. LPS-Driven Inflammatory Pathways

LPS activates inflammation through multifaceted mechanism [56, 57] Maternal LPS triggers embryonic resorption through strong cellular network which is responsible for increased excessive placental TNF-*α*, IL-1*β*, and IL-6 expressions that subsequently reduced phosphorylated Akt protein (serine/threonine-specific protein kinase) thereby causing decreased number of live pups, fetal weight, and placental weight [6, 58, 59]. Moreover, LPS also stimulates both transcription factors such as MAP kinases (MAPKs) and nuclear factor-*κ*B (NF-*κ*B) [60]. Prevalence of uterine inflammation is a major outcome of infection and idiopathic preterm birth [61] caused by alleviation of cytokine activity before preterm labor, cervix and fetal membranes by neutrophils and macrophages [62]. Several studies reported that proinflammatory cytokines such as IL-1*β*, IL-6, and TNF-*α* may activate contraction-associated proteins (CAPs) comprising oxytocin receptor (OTR), connexin 43 (CX43), prostaglandin H synthase- (PGHS-) 2, and prostaglandin receptors, in the myometrium, which exert uterotonic factors such as PGs that induce subsequently labor and inflammatory signals, suggesting a potential target in attenuating preterm birth [63]. In addition, normal labor in mouse associates with subsequent stimulation of NF-*κ*B and AP-1 within the uterus, whereas LPS-induced preterm labor (PTL) in two mouse models has been reported to have activated NF-*κ*B and Jun N-terminal kinase (JNK) transcription factors [64].

## 5. Pregnancy-Related Disorders and Adverse Birth Outcomes

### 5.1. Effects of LPS on Decidual Cells

The vast literature has been published on decidual cells, which focuses on pregnancy recognition, fetal growth, and survival. Decidual cells are the maternal tissue which acts under the influence of progesterone and testosterone in circulation in order to maintain growth following implantation of blastocyst with the endometrium. Later on, decidual and trophoblastic cells form the placenta of maternal portion [65]. Crosstalk between LPS and Toll-like receptor 4 (TLR4) resulted in harmful effects on pregnancy through releasing a variety of inflammatory cytokines in murine models [66]. Certain cytokines such as IL-4, IL-6, and IL-10 elicit beneficial effects on pregnancy [67]. Wang et al. [68] demonstrated that baicalin treatment at 4 *μ*g/mL to uterine decidual cells which was cultured with LPS on day 6 of pregnancy. Meanwhile, in *in vivo* experiment, LPS was inducted at day 6 of pregnancy and subjected on oral doses of baicalin at day 7 and day 8 of pregnancy. The results documented that baicalin prevents damage to decidual cells and reduces TNF-*α* activity, hence producing fruitful effects on pregnant mice.

### 5.2. Maternal LPS-Mediated Teratogenicity

Some studies have found that LPS induces teratogenicity by overriding of free radicals. The subcut induction of LPS causes fetal malformation such as anencephaly and eye deformities [69] and developmental toxicity regulated by maternal side [70]. Uprising of tumor necrosis factor-alpha (TNF-*α*) in fetal liver and brain-induced fetal death occurs through either maternal circulation or amniotic fluid which mediated LPS induction [71]. In addition, LPS also induced lipid peroxidation and GSH depletion in maternal liver and placenta and increases expression of HO-1 in fetal liver that was counteracted by radical trapping agent N-tertiary-butylnitrone (PBN), a compound used for spin trapping. It has been well characterized that ROS are unstable reactive species which could not be eliminated successfully during organogenesis process and transfer from maternal to fetal tissues, irrespective of avoiding antioxidant defense. Hence, lacking of GSH proclaimed to develop ROS within fetal tissues. ROS developments in fetal tissues are not well clarified [72] though TNF-*α* can cross maternal serum and amniotic fluid to fetuses [71].

### 5.3. Oxidative Stress and Preterm Birth

Premature birth frequently occurs prior to normal delivery, when antioxidants could not be so active to alleviate oxidative stress resulting in preterm birth. It develops due to hindrance in uteroplacental transfer of nutrients which keeps newborns more sensitive against increasing ROS insults [73]. The MnSOD mRNA seems to be present in fetal membranes after labor and show its existence in chorioamnionitis [74]. It has been revealed that inflammation might be involved in placental antioxidant system which depends upon the concept development. Recently, studies rectified that [75, 76] cytokines are the main regulators for premature birth; hence, expression of NF-*κ*B induced cytokines as a novel target for alternative therapeutic options. NF-*κ*B is recognized in the induction of proinflammatory genes and mediates the expression of adhesion molecule, chemotactic factors, and acute phase proteins. The activation of NF-*κ*B signaling pathway may enhance synthesis of proinflammatory cytokines that induce infected preterm birth [77, 78]. The current study has shown that polyphenols particularly curcumin exert beneficial effects on inhibition of NF-*κ*B-linked pregnant tissue-infected premature birth in mice, suppress TNF-*α* and IL-8, and mitigate oxidative insult in mothers and fetuses [79].

### 5.4. Preeclampsia and Oxidative Stress

Preeclampsia seems to be reported after 20 weeks of gestation in humans [80]. Some literatures build up strong relations between maternal inflammation and oxidative stress. Researchers stated that increased maternal inflammation through a variety of signaling pathways and presence of oxidative stress might be the possible factors for inducing preeclampsia condition [81, 82]. In preeclampsia, reactive oxygen species initiates apoptosis of syncytiotrophoblast in placentation mechanism and affects anterior remodeling [83]; hence, it mediates inflammation. In addition, oxidative stress has been presumed to stimulate maternal endothelial cells as an inducer of preeclampsia condition [84].

### 5.5. Oxidative Stress-Induced IUGR Complications

Liu et al. [85] revealed that LPS induced intrauterine growth restriction in late gestation mice. It is stated that fetal IUGR is more susceptible in late gestation to increased risk of metabolic disorders such as insulin resistance, diabetes mellitus, obesity, hypertension, and cardiovascular diseases in model animals [86, 87]. Moreover, maternal protein restriction during pregnancy triggers fetal IUGR after prompt growth and alters gene expression in adipose tissue which is more prone to obesity in adult mice [88]. Numerous literatures established links of IUGR with oxidative stress. In IUGR pregnancies, oxidative stress markers such as MDA and protein oxidation of mother and fetus erythrocytes confirmed the strong relations [89]. In addition, oxidative/antioxidant markers were elevated in IUGR pregnancies, suggesting that neonates with IUGR elicited low level of antioxidant defense lipid peroxidation [90].

## 6. Significant Impact of Nrf2 Pathway on Pregnancy

Nrf2 is a leucine-based zipped transcription factor which displays a key role against oxidative stress by induction of phase II antioxidant enzymes [91]. Activation of Nrf2 is crucial for ameliorating oxidative stress-mediated cellular damage via protection through 20S proteasome or [92] by p62-dependent autophagy [93]. Normally, Nrf2 is located in Kelch-like ECH-associated protein-1 (Keap1) [94]. Keap1 functions as sensors for oxidative stress [95]; upon activation, Nrf2 binds with Maf recognition/antioxidant response element and electrophilic response element (ARE/EpRE) in promoter target genes encompassing NAD(P)H:quinone oxireductase 1 (NQO1) [96], heme oxygenase1 (HO-1) [97], glutamate cysteine ligase(GCL) [98], and the light chain of the amino acid cystine-glutamate exchanger (xCT) [99] involved in glutathione biosynthesis. Notably, more than hundred genes have been identified; many of them are redox-sensitive transcription factors [100].

Numerous reports were described the protective effects of Nrf2 on the embryo against adverse effects of oxidative stress in utero (Table 1). Nrf2 knockout mice were considered as indicators of placental oxidative stress which suppress fetal growth [101]. Nrf2-deficient mice are vulnerable to methamphetamine-induced fetal DNA insult and neurological deficits [102], whereas polyphenols such as hydroxytyrosol-induced Nrf2 stimulation ameliorate oxidative stress-mediated effects in cognitive function and neurogenesis in offspring [103]. Activation of Nrf2 has exhibited to reduce Et-OH-induced neural crest apoptotic cells in a fetus [104], and trophoblastic triggered apoptosis by inflammation [105]. At the same time, aforementioned studies indicated that Nrf2 exerts protective effects towards oxidative insult during early-pregnancy development (i.e., neutral crest formation), while some other studies documented significant effects of Nrf2 in redox mechanism in later-developmental phases. The in utero Keap1/Nrf2 signals have been demonstrated in response to amniotic fluid through increased expressions of genes contributed in epidermal development [106]. The Nrf2 is very sensitive to the maternal immune system to mediate the function of fetal membranes to birth. Importantly, Nrf2 protein expression was decreased in fetal membranes during pregnancy due to amniotic infection. The pitfalls in Nrf2 regulation can facilitate preterm delivery; knockdown of Nrf2 in amniotic cells causes upregulations of proinflammatory cytokines which causes rupturing fetal membranes. Moreover, a beneficial effect of Nrf2 on antioxidant mechanism is more obvious in alleviating adverse developmental outcomes. In neural crest cells, where excessive glucose declines, CuZnSOD, MnSOD, catalase, GPx1, Nrf2, and Pax3 expressions induce vulnerability to these cells which leads to oxidative damage [107]. Importantly, overexpression of catalase enhances Nrf2 and its downstream HO-1 expression, thus showing a protecting role in obesity-induced diabetes fetal renal damage [108]. The Nrf2 expression is also decreased in IUGR placenta [109]. In preeclampsia pregnancies, the role of Nrf2 has been reported to be somehow controversial, whereas reduced expression of Nrf2 was noted in placental oxidative stress-induced preeclampsia [110]. Inappropriate regulation of Nrf2-based HO-1 expression mediates soluble fms-like typrsine kinase-1 (sFlt-1) [111]. Increased level of sFtl-1 has been recorded in the pathogenesis of PE and development of maternal hypertensive condition during pregnancy. Overall, Nrf2 function in normal pregnancy is incomplete although it is providing protection during uterofetal life against a variety of stressors.

Cheng et al. [112] have demonstrated that protein levels were significantly affected during redox status of GDM due to overproduction of superoxide radicals, protein oxidation, DNA damage, and reduced GSH synthesis. Moreover, in GDM cells, lipid peroxidation did not show Nrf2 genes and protein levels to its targeted genes NAD(P)H:quinone oxidoreductase 1 (NQO1), Bach1, cystine/glutamate transporter, and glutamate cysteine ligase. Lipid peroxidation triggered GSH and NQO1 activity which was revoked by Nrf2 in normal cells, and overexpression of Nrf2 in GDM cells partly restored NQO1 induction. Improper functions of Nrf2 in fetal endothelium increased the risk of inducing T2DM and CVD diseases to offspring. Zheng et al. [113] revealed the alternation in spontaneous activity and impair in learning and memory levels in prenatal stress male and female offspring. The stress was found to be due to downregulating of neuronal proteins and glucocorticoid levels. Similarly, alteration in protein oxidation, SOD, and mitochondrial activity was also declined, whereas hydroxytyrosol (HT) enhanced FOXO1 and FOXO3, Nrf2, and HO1 proteins and restored mitochondrial functions. It indicates that HT is a potential maternal nutritive compound that provides protection towards neurogenesis and cognitive offspring. In a study documented by Wan et al. [114], exhibiting the overexpressions of GSTP was contributed with transcription factors Keap1-Nrf2/MafK. Therefore, early induced alternations in cytosines within GSTP gene were referred as a biomarker of hepatic PFOS, whereas the direct role of PFOS-induced hepatotoxicity needs to be further elucidated. In another findings demonstrated by [115], it was shown that hyperoxia induced alveolar growth in neonatal lung by induction of p21/p53 pathways, a potential risk for developing bronchopulmonary dysplasia (BPD) in preterm infants. Results indicate that activation of Nrf2 pathway promoted antioxidant response genes which were declined by hyperoxia. Dong et al. [116] reported that exposure of maternal ethanol induces fetal alcohol syndrome that enhanced expression of Nrf2 and Nrf2-ARE protein levels in mouse embryos. Hence, it increases the response of proteins and antioxidant enzymes. In addition, dithiole-3-thione (D3T) treatment minimizes ethanol-mediated reactive oxygen species productions and inhibits apoptosis in mouse embryos. The results reported that simulation of Nrf2 was involved in releasing antioxidant response against exposure of ethanol embryos. In other investigation, it was documented that H_2_O_2_ decreased glutathione peroxidase (GSH), thioredoxin-1 (Trx1), and mitochondrial thioredoxin-2 (Trx2) in a whole cultured embryo with 10 *μ*M dithiole-3-thione (D3T). D3T enhanced Nrf2 responsive genes. These findings showed that stimulation of Nrf2 provides protection against chemically mediated oxidative stress by maintaining intracellular redox mechanism, thereby stabilizing normal embryo development [117].

## 7. Dietary Supplementation of Polyphenols during Pregnancy

Flavonoids, the compounds of polyphenols, have received worldwide recognition due to their enormous existence in nature, and more than 10000 diverse molecular components have been identified so far [118]. Foods, vegetables, fruits, and herbs are rich sources of flavonoids [119]. It has come into central position due to presenting several functions encompassing antioxidant, anti-inflammatory, and antiabortogenic properties [120, 121]. LPS mediates inflammation through numerous series of cellular events that subsequently stimulates NF-*κ*B pathway which encodes genes for inducing inflammation such as iNOS, NO, and COXs that synthesize prostaglandins and cytokines. Moreover, Toll-like receptors are responsible for the production of reactive oxygen species [122, 123]. As described above, LPS mediates pregnancy disorders and adverse birth outcomes through the regulation of proinflammatory cytokines such as TNF-*α* and IL-8 in maternal sera, amniotic fluid, fetal liver and fetal brain [124] and induced fetal IUGR, fetal resorption, and preterm delivery which was reversed by TNF-*α* inhibitor and chemokine inhibitor, respectively. Flavonoids suppress chemokine production comprising TNF-*α*, IL-1*β*, and monocyte chemoattractant protein-1 [125]; some protective effects of polyphenols are illustrated in Table 2.

The uptake of enriched polyphenol food has been documented to enhance plasma antioxidant status in humans [126] and reduce incidences of oxidative insult *in vitro* and *in vivo* studies in a human placenta and trophoblasts, respectively [127]. The flavonoids ameliorate oxidative stress-mediated inflammatory response by suppression of inflammatory mediators (reactive oxygen species (ROS) and nitric oxide (NO)), decreased inflammatory enzymes such as cyclooxygenases (COXs) and inducible nitric oxide synthase (iNOS) modulating NF-*κ*B and activating protein-1 (AP-1) signals [26, 128] decreasing cytokine expressions, and activation of phase II enzymes glutathione S-transferase (GST) [129]. Supplementation of polyphenols has shown beneficial effects on pregnancy and was referred as therapeutic intervention to encounter pregnancy disorders and adverse birth outcomes [130]. Lack of antioxidant defense creates hindrances in homeostasis due to the exceeding amount of ROS, while their supplementation may show protective effects [130].

**Figure 2:**
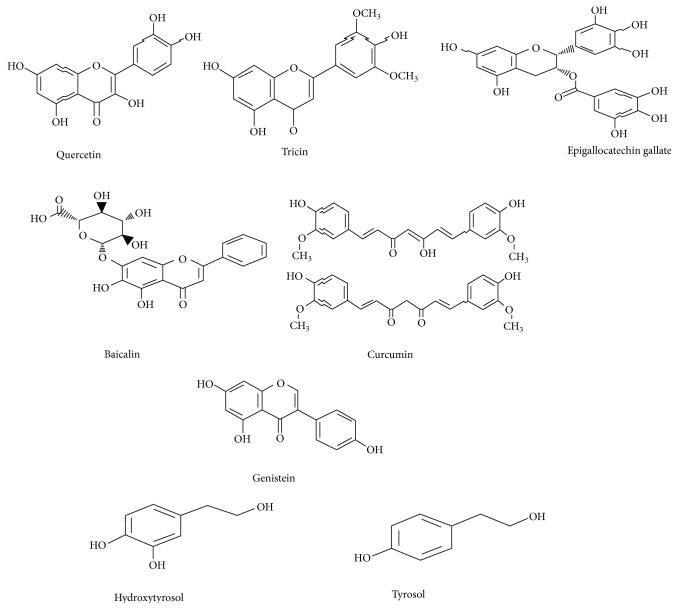
Some polyphenol compounds and their chemical structures.

Vanhees et al. [131] exhibited that exposure to intrauterine flavonoids such as quercetin and genistein at lower level inhibited oxidative stress and DNA damage in the liver of adult mice that subsequently develops antioxidant environment through regulation of Nrf2 signaling pathway. It indicates that dietary antioxidant supplementation during gestation develops long lasting antioxidant defense that eliminate oxidative stress at adulthood, where oxidative stress was assumed to be involved in chronic diseases. Importantly, LPS-mediated inflammation plays a key role in embryonic resorption, fetal growth restriction, and preeclampsia [132]. The polyphenols like curcumin ameliorate abnormal pregnancy outcomes by suppressed LPS-triggered inflammation in mice. The anti-inflammation activity of curcumin was achieved by upregulation of phosphorylated Akt pathway which was decreased by LPS induction [59]. Tricin, a polyphenol-derived compound, encountered inflammation by activation of Akt signals and cellular proliferation. This anti-inflammatory effect of Akt pathway was obtained by inhibition of IKK protein activity which brings NF-*κ*B back into the cytoplasm in its normal physiological position [133]. Several compounds like the flavonoid group of polyphenols induced stimulation of Nrf2 signals. This evidence was proved by [134] who revealed the neuroprotective properties of polyphenols by activation of Nrf2/HO-1, thereby exerting therapeutic insights of polyphenolic compounds. Another example of epigallocatechin gallate (EGCG) treatment enhanced nuclear accumulation, anti-oxidant response element (ARE) binding with Nrf2.These results indicated that ECGC regulated Nrf2-mediated expression of few antioxidant enzymes particularly stimulation of Akt and ERK1/2 signaling; hence, it supports antioxidant system in attenuating oxidative stress [135]. Some polyphenols and their chemical structure are depicted in Figure 2. Literature has shown a less number of studies on antioxidant supplementation such as flavonoids during pregnancy, as it was known as strong antioxidative compounds proven from other evidence, whereas some report exhibited ambiguous results that might be due to timing of supplementation, improper dose regimen, and lack of antioxidant-targeted compounds. More research should be warranted to explore methods for minimizing uterine oxidative stress and mimic ROS-mediated diseases from mothers to newborns.

## 8. Concluding Remarks and Future Perspectives

We have clearly defined that stimulation of Toll-like receptors by LPS-induced generation of free radicals and their excessive amount leads to fetal resorption, preterm birth, teratogenicity, intrauterine growth restriction, abortion, neural tube defects, fetal demise, and skeletal development retardation. Moreover, oxidative stress also activates NF-*κ*B, PPAR-*γ*, AP-1, and JNK pathways which accounts for pathological conditions in aforementioned pregnancy disorders. In addition, NF-*κ*B is responsible for transcription of several proinflammatory cytokines which are known to induce pregnancy disorders and adverse outcomes such as TNF-*α*, IL-1*β*, IL-6, and PGF2E. Importantly, stimulation of Nrf2 signals plays a crucial role in ameliorating pregnancy insults. It was further counted that oxidative stress is the major contributing factor, whereas polyphenols are the novel compounds for treating oxidative stress-related disorders. Limited studies have been documented on polyphenol supplementation during pregnancy and its outcomes. It was presumed that intrauterine fetal life decides the future of a wide array of complications which appear at later stages of life. Nutrition and antioxidant supplement are the main players for fetal reprogramming. Any impairment in this system might have disturbance in extrauterine life. Studies reported that strong maternal uterine antioxidant environment could prevent pregnancy disorders and abnormal birth outcomes and could also prevent other complications later in life which might initiate from embryonic stage. More molecular evidences are required for antioxidant/inflammatory events from fertilization to parturition during pregnancy. We assume that these findings would be helpful in understanding oxidative stress-induced pregnancy insults and might give new roadmap to researchers for therapeutic intervention which could subsequently improve human and animal fertility.

## Figures and Tables

**Table 1:** Some enlisted Nrf2 gene regulation in maternofetal tissues.

Origin	Regulation of Nrf2 protein/gene	Functional significance	References	
Human umbilical endothelial cells	NQO1, GCLM, Nrf2, GSK3*β*	GDM ↑ oxidative stress and ↓ Nrf2 activity and overexpression of antioxidant expression	[112]	
Rat	Nrf2, HO-1, SOD2	Hydroxytyrosol (HT) and moderate Restraint stress (GD14-20) ↑ Nrf2-dependent gene expression	[113]	
Rat liver	GSTP, Nrf2	Maternal perfluorooctane sulfonate ↑ methylation of Nrf2-dependent GSTP gene promoter	[114]	
Nrf2^−/−^ and WT mice	Nrf2, GSTA3, MGST1, GSTA4 Gpx2, AKR1B1, AKR1B10, NQO1	Postnatal hyperoxia ↑ Nrf2-dependent gene expression, abolished in Nrf2^−/−^ mice	[115]	
Mouse embryos	Nrf2, SOD1, SOD2, SOD3, CAT, Trx, Gpx1, Gpx2, Gpx3, GR	Maternal ethanol or D3T exposure ↑ Nrf2-dependent gene expression	[116]	
Mouse embryos	GSH, NQO1, HO-1, GCLC, GST, Prx1 Trx1, Trx2	Maternal D3T administration ↑ Nrf2-dependent gene and ↓ H2O2-induced Trx1 and Trx2 oxidation	[117]	

AKR1B1: aldo-keto reductase family-1 member B1 (aldose reductase); AKR1B10: aldo-keto reductase family-10 member B10 (aldose reductase); CAT: Catalase); GCLC: glutamate-cysteine ligase catalytic subunit enzyme; GCLM: glutamate-cysteine ligase regulatory subunit enzyme; GDM: gestational diabetes mellitus; GR: glutathione reductase; GSK3*β*: glycogen synthase kinase 3 beta; GSH: glutathione peroxidase; GSTA3: glutathione S-transferase alpha-4; GSTA4: glutathione S-transferase alpha-4; Gpx1, 2, and 3: glutathione peroxidase 1, 2, and 3; GST: glutathione S-transferase; GSTP: glutathione reductase; GPO: glutathione peroxidase; HO-1: heme oxygenase; MGST1: microsomal glutathione S-transferase 1; NQO1: (NAD(P)H:quinone dehydrogenase 1; Nrf2: NF-E2-related factor 2; Prx1: peroxiredoxin 1; SOD1, 2, and 3: sodium dismutase 1, 2, and 3; Trx1 and 2: thioredoxin-1 and 2).

**Table 2:** Beneficial effects of polyphenols in LPS-induced pregnancy disorders.

LPS doses	Gestation stages (days)	Pregnancy disorders	Flavonoids/protective effects	References
LPS 0.2 mL/0.2 *μ*g/mouse	4–7	Abortion	Quercetin indicates antiabortive effects through influence on CD4+/CD8+ T lymphocytes and IFN and IL-4	[136]
LPS 0.1 *μ*g per mouse	6.7	Fetal resorption	Polyphenolic compounds of Radix Scutellariae and Rhizoma Atractylodis (baicalein, wogonin, oroxylin, baicalin, wogonoside, oroxylin A-7-glucuronide reduced fetal resorption and including IL-10 Pharmacological effects and pharmacokinetic properties of Radix Scutellariae and its bioactive flavones	[137, 138]
LPS, 0.1 mL/10 g *in vitro/in vivo*	6 6 & 7	Injury of decidual cells	Baicalin, 4 *μ*g/mL *in vitro* and *in vivo* at different doses prevents decidual cell injury by inhibition of TNF-*α*	[68]
LPS at 0.2 ml, murine model	7	Abortion and reabsorption	Bao Tai Wu You, Tai Shan Pan Shi, or Bai Zhu San at 0.5 ml oral medication for 7 days ameliorates INF-*γ* and increases IL-10 and IL-4 thus showing beneficial effects	[139]

CD4 and 8: cluster of differentiation 4 and 8; IFN: interferon; IL-4: interleukine-4; IL-10: interleukine-10; INF-*γ*: interferon gamma; TNF-*α*: tumor necrosis factor-alpha.

## Abbreviations

Akt: Serine/threonine-specific protein kinase
AP-1: Activated protein kinase-1
CAPs: Contraction-associated proteins
CD14: Cluster of differentiation 14
COX-2: Cyclooxygenases-2
CX43: Connexin 43
EGCG: Epigallocatechin-3-gallate
ERK1/2: Extracellular signal-regulated kinase 1/2
ESC-B5: Embryonic stem cell B5
FGR: Fetal growth rate
FOXO1 and 3: Forkhead box protein O 1 and 3
GCL: Glutamate cysteine ligase
GDM: Gestational diabetes mellitus
GST: Glutathione S-transferase
HO-1: Heme oxygenase1
IFN*γ*: Interferon-*γ*
iNOS: Inducible nitric oxide synthase
IUGR: Intrauterine growth restriction
JNK: Jun N-terminal kinase transcription factors
LPS: Lipopolysaccharides
MAPKs: Mitogen-activated protein kinases
MDA: Malondialdehyde
MYD88: Myeloid differentiation factor
NF-*κ*B: Nuclear factor kappa B
Nrf2: NF-E2-related factor 2
OTR: Oxytocin receptor
NO: Nitric oxide
PE: Preeclampsia
PGs: Prostaglandins
PGHS: Prostaglandin H synthase
PPAR*γ*: Peroxisome proliferator-activated receptor gamma
ROS: Reactive oxygen species
sFlt-1: Soluble fms-like typrsine kinase-1
SOD1: Sodium dismutase-1
TNF-*α*: Tumor necrosis factor-*α*
uNK: Uterine natural killer
xCT: Cystine-glutamate exchanger.
